# STEMI time delays: a clinical perspective: Editorial comment on the article by Verweij et al.

**DOI:** 10.1007/s12471-015-0728-z

**Published:** 2015-07-18

**Authors:** M-J. de Boer, F. Zijlstra

**Affiliations:** 1 Department of Cardiology, Radboud University Medical Center Nijmegen, Geert Grooteplein 10, 6525 GA Nijmegen, The Netherlands; 2 Department of Cardiology, Erasmus Medical Center Rotterdam, Rotterdam, The Netherlands

**Keywords:** Primary angioplasty, STEMI, Time delay, Mortality, Morbidity

## Abstract

STEMI time delays have been introduced as a performance indicator or marker of quality of care. As they are only one part of a very complex medical process, one should be aware of concomitant issues that may be overlooked or even be more important with regard to clinical outcome of STEMI patients. In this overview we try to summarise the most important ones.

## Introduction

**Fig. 1:**
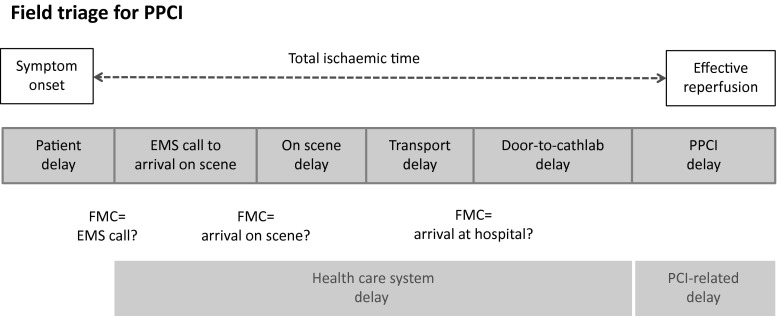
Various delays when treating patients with ST-Elevation Myocardial Infarction (STEMI) with primary percutaneous coronary intervention (PPCI). ‘Healthcare system delay’ is the total delay from emergency medical service (EMS) call to PPCI. ‘PCI-related delay’ is the extra delay that one may use to perform PPCI and achieve effective reperfusion. First Medical Contact (either EMS call, EMS arrival on scene, or arrival at hospital according to regional STEMI system of care, after reference 15)

Maybe the most important step forward in cardiology care is the early treatment of acute ischaemia of the heart. By performing immediate angiography in patients with the clinical presentation of an acute coronary syndrome (ACS), and more specifically when there are signs of ST elevation on the 12-lead ECG (ST-segment-elevation myocardial infarction: STEMI), subsequent percutaneous coronary intervention (PCI) to restore blood flow in blocked coronary arteries will save lives [1–3]. By improving the logistics surrounding these procedures, better results in outcome may be accomplished. Since mortality and morbidity increase with time without treatment, STEMI protocols are applied, with different methods and techniques to shorten ischaemic time delays [3, 4]. This involves many steps in a chain of ambulances, pre-hospital triage and treatment strategies, dedicated cardiac emergency units, ancillary paramedic personnel and additional measures, including monitoring of care for optimal quality. Furthermore, there is a strong relation between volume and outcome and nowadays it is widely recognised that a highly trained experienced team treating STEMI patients performs better [5, 6]. This can be measured by mortality and morbidity, but also by monitoring and comparing weaker endpoints as hospital stay, quality of life, preservation of left ventricular function, readmissions, the need for re-interventions, and many more [6]. A network-oriented approach with triage and diagnosis by paramedics in the ambulance will bypass non-PCI centres in favour of PCI capable centres, and may thus further reduce time delays [7, 8]. If timely PCI cannot be accomplished, fibrinolytic therapy should be considered but there still is a large debate on the time delay that is acceptable, before deciding for this option, or when a combination of the two reperfusion modalities should be preferred [9–11]. In daily practice in the Netherlands, practically all STEMI patients can be transported to a dedicated PCI catheterisation lab within 60 min after first medical contact (FMC), usually the time of the emergency call [8, 12]. In this issue of the journal, Verweij et al. try to compare data on time delays and timing of intervention in different hospital settings in the Netherlands and their conclusion is that a lot of data are inconsistent and/or incomplete or even missing or incorrect [13]. Of note: a correct registration of data is a prerequisite for having a license to perform interventional cardiology in the Netherlands. This observation deserves a careful analysis. First there is a variation in definition in the international guidelines and in the literature in general [14, 15]. This, in part, stems from a more regional than international perspective but still accounts for much confusion. For instance, there is no universal definition of FMC, as is illustrated in Fig. 1. Second, in the Netherlands we regretfully still do not have a uniformly organised national cardiology data registry, such as in the UK or Sweden (Swedeheart)[16], or the National Cardiovascular Data Registry (NCDR) in the US. This, in our view, deserves the highest priority of all stakeholders involved but is a political issue outside the context of this article.

## Definition of STEMI time intervals

The most commonly used parameters or indicators are the time from symptom onset till effective reperfusion (total ischaemic time) and the median door-to-balloon time (D2BT), the latter supposed to represent the in-hospital performance. Besides, D2BT is easily determined and it was quickly introduced as a measure of quality of care of STEMI patients. The mean D2BT in our first study group of primary PCI patients in the early 1990s was only 61 min, although this concerned patients who were also eligible for fibrinolytic therapy [2]. Other issues sometimes addressed are: call-to-balloon time (CTB), time from FMC, time from first ECG, time to the catheterisation lab, time to coronary angiography, door-1 to door-2 time (D1D2 time) in case of inter-hospital transfer, and so on. All have their strong and weak points and often have to be registered in hectic situations, and during off-duty hours. For a comprehensive overview of system delays in primary angioplasty field triage, we refer to Fig. 1. Furthermore, different hospitals use different electronic health records, with different databases, or still have to operate without automated system files. The pre-hospital phase data, mostly from ambulances, are usually stored separately. This inevitably accounts for the lack of consistency of methods and data registration and makes comparison among and between hospitals and/or health care providers difficult. However, we need these data to improve our performance and ability to monitor all aspects of this complicated chain of medical care, and to find ways for improvement, not only of the PCI procedure itself, but also its surrounding logistics. Our suggestion would be to count system time delay from first ECG to the time of start of coronary angiography (CAG), both being easily obtainable and above all, automatically stored in current practice. As primary angioplasty is the preferred treatment in almost all candidates identified by pre-hospital triage or by direct presentation to hospitals, we should not use the term ‘door-to-needle time’ anymore, as was used in previous studies and surveys that concerned mainly patients who received fibrinolytic therapy [17].

International comparisons have demonstrated significant differences in infarct care organisation and outcomes between countries, and national surveys have proven to be useful in improving the system and quality of care in STEMI patients [16, 18]. In many countries a substantial number of STEMI patients are not receiving any reperfusion therapy at all or receive it outside the guideline-recommended timeframes, and implementation of the best reperfusion therapy as recommended in the guidelines should be encouraged. This includes efforts at reducing D2BT for STEMI patients undergoing primary angioplasty, regardless of the clinical setting or health care region.

In general, guidelines and quality-of-care programs for patients with STEMI arbitrarily recommend a D2BT of less than 90 min. for primary PCI.

## Important issues

There are important issues that should be addressed in relation to interpretation of STEMI time delays but are usually not taken into consideration:

- Sicker patients and patients with high-risk features undergo more delay [19]. There may be large differences in baseline characteristics in the patients studied. Prognostic factors including age, comorbidities, presence of diabetes, previous myocardial infarction or congestive heart failure, haemodynamic state on admission and infarct size are usually not reported but may result in differences in outcome [18, 20]. Above all, if adjustment for these characteristics is applied, time delay does not seem to be a significant determinant of outcome anymore.
- Despite reductions in D2BT, there has been little change during the past few years in in-hospital mortality, suggesting that additional factors play an important role [21].
- Outcome and time delays may be related to the presentation of STEMI patients during off-duty hours. Patients with acute myocardial infarction who present during off-hours have a higher mortality than those who present during regular hours and a recent meta-analysis suggests that this is not directly related to total ischaemic time delays [22, 23]. However, the average D2BT appeared to be longer in off-hours and this may be an expression of variations in quality of care (number and expertise of staff, and other structural and process attributes in systems of care).
- High versus low volume centres: volume and outcome may translate into different outcomes and sicker patients may be presented more often to large volume centres [5, 6].
- Operator skills, mode of vascular access (femoral versus radial route), procedural time, availability of advanced technology and surgical stand-by may all play an important role and are usually not included as a variable, when it comes to comparison of outcome [12].
- Despite the shortest time delay, patients with out-of-hospital cardiac arrest (OHCA) have the highest mortality [19]. We need more data on the growing number of OHCA patients who are candidates for primary PCI and because OHCA is usually related to cardiac arrhythmias on the basis of cardiac ischaemia, dedicated cardiac resuscitation centres have been proposed with promising results [24, 25]. These ideally should be equipped with 24/7 primary angioplasty facilities, include therapeutic hypothermia capability and a specialised intensive care unit, fulfilling requirements for optimal post-resuscitation care [26].
- Currently a debate is going on whether all OHCA patients should be scheduled for immediate CAG, regardless of whether they have features of STEMI. However, the net clinical benefit of emergency CAG in comatose OHCA survivors can only be assessed in prospective randomised studies, but as mortality in these patients is extremely high, efforts to improve survival and neurological outcome deserve the highest priority.
- By focusing too much on the reported criteria—especially using time relapsing after FMC as an indicator of performance—we may miss an important moment of contemplation. Is the diagnosis correct? Is there additional pathology, for instance neurological trauma, renal impairment or a bleeding disorder? Should a CT scan of the brain be performed first? Are the other supportive measures of vital functions sufficient? Should mechanical complications be ruled out? Is cardiac surgery a better option and should we ask the cardiothoracic surgeon’s opinion first? This is more difficult in sicker patients, and it may take longer to decide on the right therapy [15, 27].
- Following the abovementioned reasoning, it does not seem justified to correct for anticipated time delays or to try to estimate the total ischaemic time for making the decision to proceed or not proceed to an invasive procedure. This may lead to an incorrect approach, especially in sicker patients and those with high-risk features for mortality. If time delay is very long or if there is uncertainty about total ischaemic time it may even lead to the decision not to intervene at all. As this is usually the case in sicker patients, who might have benefited the most, this may be an unwanted side effect of using time delays as a performance indicator [28].
- Although it seems reasonable to focus on system delays, the detrimental effects in the first hours may be overestimated, and the phrase ‘every minute of delay counts’ should be brought into perspective. Careful calculation shows that each 30 min of delay is associated with a relative risk for 1-year mortality of 1.05–1.07 [3, 29–31] However, this, in our view, in general allows for transportation of stable STEMI patients to an experienced interventional centre and to think about correctness of diagnosis and the best treatment option in individual cases, as was mentioned above. Opportunities for optimal STEMI patient care may be missed and patients may undergo an inappropriate early intervention.
- The currently established pathways of transfer of STEMI patients in the Netherlands are not likely to be improved by emphasis on time delays alone, and the impact on outcome can certainly not be investigated in a randomised comparison. The scope of cardiologists in STEMI treatment mainly puts the focus on shortening the time to treatment after the patient has arrived at the hospital. However, a large proportion of STEMI-related death has occurred before arrival at the hospital.

## Conclusion

Shorter time delays will not necessarily translate into better outcome and this puts time delay as performance indicator into another perspective [19]. Of course initiatives and programs to reduce time delays have been successful and are needed, but we definitely should not promote them to the ultimate goal. By putting the focus of STEMI care too much on the simple comparison of time delays suggests that good clinical care can be data driven and a comparison with cholesterol-lowering drugs comes to mind: a 10 % drop in LDL cholesterol does not necessarily mean a reduction of 10 % in adverse outcome.

Clinicians are treating sick patients and should realise that they do not perform well by producing nice indicators and criteria for hospital registrations, but by presenting patients the best treatment options available, although in a timely fashion. Reduction of time delays remains crucial in the treatment of the STEMI patient, but this is only one part of a very complex chain of medical care. Hospital administrations, health care providers and politicians should be aware of the limited value of STEMI time delays as a quality indicator. Although they are very helpful in streamlining logistic processes, they should not be considered as a proxy for hospital performance or as a surrogate marker of quality of care.

### Funding

None.

### Conflict of interests

None declared.
